# Outcome of the modified Dunn procedure in severe chronic or acute on chronic slipped capital femoral epiphysis

**DOI:** 10.1186/s13018-019-1433-1

**Published:** 2019-11-08

**Authors:** Nicola Ebert, Martin Rupprecht, Ralf Stuecker, Sandra Breyer, Norbert Stiel, Matthias H. Priemel, Alexander S. Spiro

**Affiliations:** 1 Department of Pediatric Orthopaedics, Altonaer Children’s Hospital, Bleickenallee 38, 22763 Hamburg, Germany; 2 Department of Orthopaedics, University Medical Center Hamburg-Eppendorf, Martinistraße 52, 20246 Hamburg, Germany; 3 Department of Trauma, Hand, and Reconstructive Surgery, University Medical Center Hamburg-Eppendorf, Martinistraße 52, 20246 Hamburg, Germany

**Keywords:** Modified Dunn procedure, Slipped capital femoral epiphysis, Avascular necrosis of the femoral head

## Abstract

**Background:**

In recent years, the modified Dunn osteotomy has gained popularity to treat slipped capital femoral epiphysis (SCFE) with various complication rates. Most studies included patients with different severities. This study aimed to determine (1) the radiological and clinical outcome, (2) the health-related quality of life, and (3) the incidence of avascular necrosis of the femoral head (AVN) in patients with severe chronic or acute on chronic SCFE treated by the modified Dunn procedure.

**Methods:**

Out of 150 patients with SCFE treated at our institution between 2001 and 2014, 15 patients (mean age 12.9 years (range 11.8–15)) were treated by the modified Dunn procedure. Eight SCFE were chronic and 7 acute on chronic. All slips were severe with a mean Southwick slip angle (SSA) of 67° (range 60–80). Radiographic and clinical outcomes were measured. Mean time of follow-up was 3.8 years (range 1–10).

**Results:**

Anatomical reduction was achieved in all cases. Good radiological results according to the Stulberg Classification (grade 1 + 2) and the Sphericity Deviation Score (< 30) were found in 9 out of 13 patients at the last follow-up. Clinical and functional outcome analysis revealed good results in 8 out of 10 patients (Harris Hip Score > 80). The quality of life measured by the Nottingham Health Profile (NHP) was described good in 10 out of 10 patients. Four out of 15 patients developed an AVN.

**Conclusions:**

The modified Dunn procedure has a great potential to restore proximal femur geometry in severe chronic or acute on chronic SCFE. It should be considered only if there is no other possibility to restore proximal femur geometry, as is the case in severe slips, due to the risk of AVN.

## Introduction

Up to date, there is still no consensus on how to treat slipped capital femoral epiphysis (SCFE). Complications after SCFE range from most severe avascular necrosis (AVN) of the femoral head to metaphyseal deformity which may lead to femoroacetabular impingement and cartilage as well as labral damage [1–5]. Every SCFE should be stabilized to avoid further slipping and major complications, but further treatment depends on the type of SCFE. Different classification systems describe the severity of SCFE and the risk for AVN. Three classification systems are widely accepted, based on the stability of the slip [6], the duration of symptoms [7], and the extension of the slip [8].

In the treatment of acute, unstable slips, when weight-bearing is not possible, there is an agreement that SCFE has to be surgically treated as an emergency case within 24 h [6, 9, 10]. Whether additional hip decompression is a protective factor against AVN is not clear [11, 12].

In stable and chronic SCFE the treatment depends on the severity of the slip. For mild slips (Southwick angle < 30°) in situ pinning as final treatment has a wide acceptance as the prognosis, and the potential for remodeling of the metaphyseal deformity is believed to be good [13, 14]. Moderate and severe slips have an increased risk of developing osteoarthritis [15]. After primary in situ stabilization, remodeling is not sufficient and residual metaphyseal deformity often leads to severe femoroacetabular impingement, cartilage as well as labral damage, and some shortening of the leg [1, 16]. Many techniques have been described to correct this residual deformity. Intertrochanteric osteotomies are often used in moderate slips, but the potential for correcting the deformity is limited especially for severe slips [17].

For severe SCFE realigning, the deformity by a subcapital Dunn osteotomy by surgical hip dislocation has gained popularity [18–21]. There is a high potential to correct the deformity, but the potential for complications is significant. Among all complications, the incidence of AVN is of particular interest. Historical studies on subcapital wedge osteotomies have reported AVN rates of up to 54% [15, 22]. The modified Dunn procedure, where the retinacular vessels are protected in a periostal flap during the reduction of the femoral head has a lower AVN rate [19]. A review by Ziebarth and colleagues demonstrated no AVN in 40 hips treated by the modified Dunn procedure in two institutions [19]. Many subsequent studies reported AVN rates between 10 and 28% [20, 23, 24]. Comparing those studies is difficult. The classification systems used to describe the type of SCFE are different or unclear. Different types of SCFE, i.e., stable and unstable SCFE were combined in one study. Many studies are multicenter studies with different surgeons and treatment algorithms.

The purpose of this retrospective study was to determine (1) the radiological and clinical outcome, (2) the health-related quality of life, and (3) the incidence of avascular necrosis of the femoral head (AVN) in patients with severe chronic and acute on chronic SCFE of > 60° treated by the modified Dunn procedure.

## Methods

### Patients

Out of 150 patients with SCFE treated at our institution between 2001 and 2014 15 patients fulfilled the inclusion criteria of having chronic or acute on chronic slips with a slip angle of > 60° being treated by the modified Dunn procedure [7, 8]. Patients who had previous surgery to correct hip deformity were excluded. All patients presented with pain and severe limping. Radiographs (anteroposterior and Lauenstein frog-leg view) were evaluated before and directly after surgery, as well as at the time of last follow-up [8]. The modified Dunn technique was elected to treat the deformities in order to restore femoral anatomy. All patients had surgery by the senior author (R.S.). Postoperative management included non-weight bearing for 8 weeks. Full weight-bearing was generally allowed after 12 weeks unless signs of avascular necrosis were detected. In these cases, non-weight bearing was recommended. Fifteen patients (7 males, and 8 females) could be included in the study. The mean age at operation was 12.9 years (range 11.8–15 years). Mean follow-up was 3.8 years (range 1–10 years). Eight SCFE were chronic and 7 acute on chronic. All slips were severe with a mean SSA of 67° (range 60–80°) (Table 1).

**Table 1:** Patient data

	Age (year)	Sex	Clas	SSA	BMI (kg/m^2^)	ToFU (year)
1	12	F	C	60°		2
2	12	M	AOC	65°	29	
3	13	M	AOC	78°	33	10
4	12.5	M	AOC	60°	34	8.6
5	13.7	F	AOC	70°	28	8
6	12.4	F	C	78°	29	8
7	13.6	M	AOC	60°		1
8	13.7	M	AOC	68°		1
9	15	M	C	80°	19	3
10	12.9	F		60°		
11	13	F	AOC	75°	31	3
12	12.5	F	C	60°	32	2
13	11.8	F		60°	22	1
14	12	M	C	70°	27	1.3
15	13.5	F	C	65°	28	1
Mean (SD)	12.9 (0.87)			67° (7.5°)	28 (4.7)	3.8 (3.4)

*Age* age at time of surgery in years, *F* female, *M* male, *Clas* Classification, *C* chronic, *AOC* acute on chronic, *SSA* Southwick slip angle, *BMI* body mass index, *ToFU* time of follow-up in years

### Surgical procedure and postoperative protocol

The stability of SCFE was evaluated during surgery in each case. SCFE was classified as unstable in case of a visible and demonstrable mobility between the metaphysis and epiphysis [25]. All patients had stable SCFE in this study. The surgical technique was according to the description of Ganz and colleagues [19]. The patient was positioned in a full lateral position, a trochanteric flip osteotomy was performed, a Z-shaped capsulotomy was done, and the femoral head was temporarily fixed with a K-wire, followed by surgical hip dislocation. The blood flow of the femoral head was tested by drilling with a K-wire before hip dislocation and after reduction in each case. The most important step was the preparation of a retinacular flap by which the retinacular vessels were protected. Further steps were the removal of the remaining physis and of abundant posterior callus. After osteotomy at the level of the physis, the head could be dissected off the femoral neck carefully, removed, repositioned, and fixed with two 6.0-mm cannulated screws. Replacement and fixation of the greater trochanter was performed with two 4.5 or 6.5 mm screws. Postoperative hip flexion was limited to 70°, and an abduction splint was used while sitting or lying.

### Outcome measurements

Assessment of radiographic outcome was done by analyzing the anteroposterior and Lauenstein frog-leg views preoperatively and at the last follow-up. The incidence of AVN, Stulberg Classification, and Sphericity Deviation Score was determined. The Stulberg Classification grades the congruency of the acetabulum and the femoral head [26]. Stulberg grades 1 and 2 are accepted to represent a good result, while grade 3 and more are considered to be non-satisfactory [27].

Sphericity Deviation Score is a measure for the sphericity of the femoral head [28]. A score up to 10 represents a very good, up to 30 a fair, and over 30 a non-satisfactory result.

The complications are listed and graded by the Dindo-Clavien Classification. Grade I complications require no treatment, grade II complications require other than usual postoperative treatment, grade III complications require surgical intervention, grade IV complications can lead to permanent disability, and grade V complication is fatal [29].

The clinical and functional outcome was assessed using the Harris Hip Score at the most recent follow-up [30]. Health-related quality of life was measured by the Nottingham health profile [31, 32]. Pain level was described by a visual analogue scale [33].

## Results

### Radiographic measurements

**Fig. 1:**
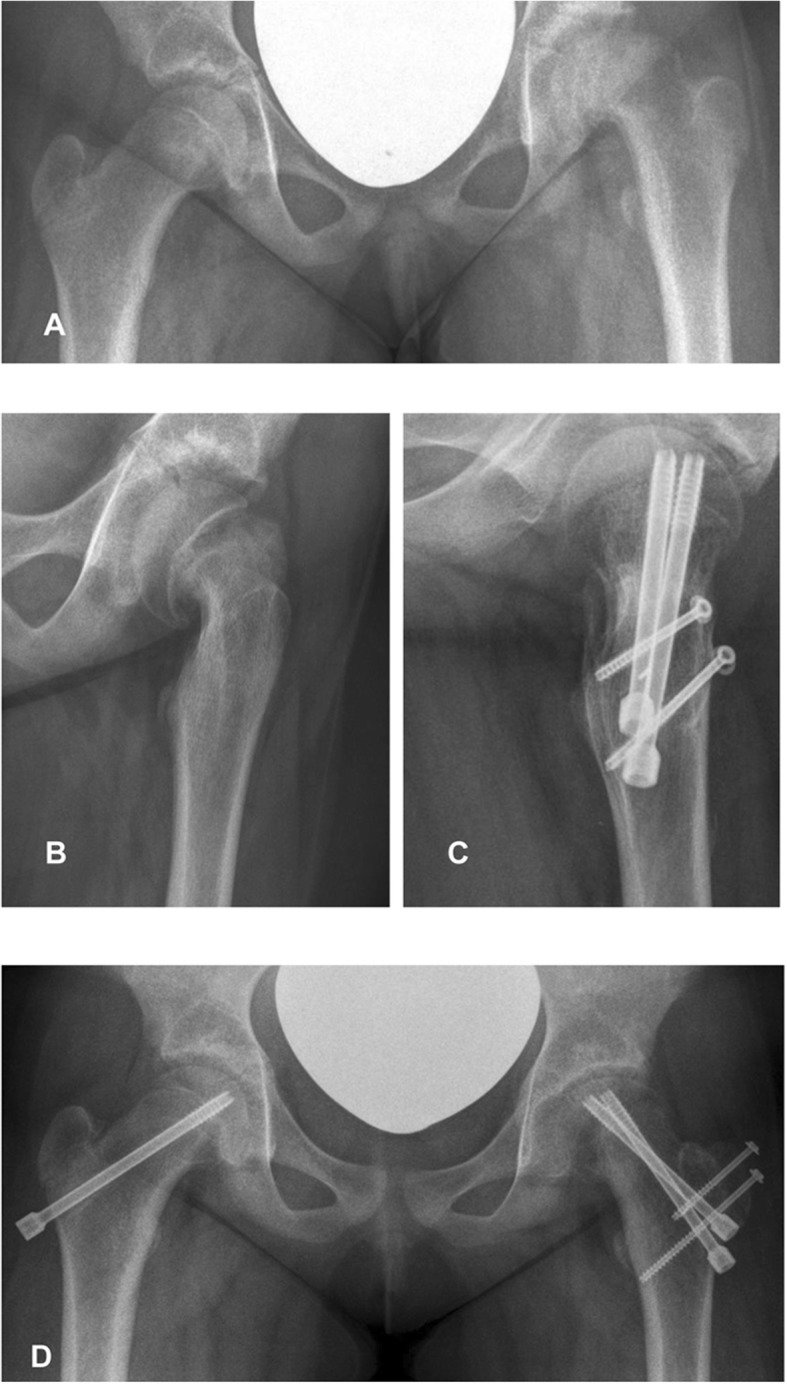
Preoperative (**a**, **b**) and 1 year postoperative (**c**, **d**) X-rays of a 13-year-old female patient with an acute on chronic SCFE with an SSA of 65°

Anatomical reduction was achieved in all cases. Two patients could not be personally examined at the last follow-up, and radiographs of those two patients were not available. However, the available radiographic reports of both patients did not describe any signs of AVN after 12 and 20 months, respectively. Of the remaining 13 patients, 4 patients had an AVN, and 69% (9 patients) had good results according to the Stulberg Classification and the Sphericity Deviation Score (Table 2) (Fig. 1).

**Table 2:** Outcome at last time of follow-up

	SC	SDS	HHS	NHP	VAS	AVN	DCC
1	2	7					0
2							III
3	5	44	58	0.97	0.8	1	IV
4	2	28	90	0.8	2		0
5	2	29	80	0.79	1.5		0
6	3	78	77	0.93	6.2	1	III
7	4	54				1	III
8	2	17					II
9	2	21	96	0.9	0		0
10							0
11	3	42	84	0.98	0.6	1	II
12	2	19	93	0.98	0		0
13	1	11	100	0.96	0		0
14	2	8	86	0.86	1		0
15	1	3	93	0.91	2		0
Mean (SD)		27.8 (21.7)	85.7 12.1	0.91 (0.07)	1.6 (1.7)		

*SC* Stulberg Classification, *SDS* Sphericity Deviation Score, *HHS* Harris Hip Score, *NHP* Nottingham Health Profile, *VAS* visual analog scale, *AVN* avascular hip necrosis, *DCC* Dindo-Clavien Classification

### Clinical outcome and health-related quality of life

Five patients were lost to clinical follow-up due to age (> 18 years of age at the time of FU) or they moved far away and were not available for further examination. One of them had an AVN. Regarding the remaining 10 patients, 8 patients (80%) had good results in the Harris Hip Score.

All 10 had good results in the Nottingham health profile. Only one out of 10 patients had relevant pain during daily activities.

### Complications

**Fig. 2:**
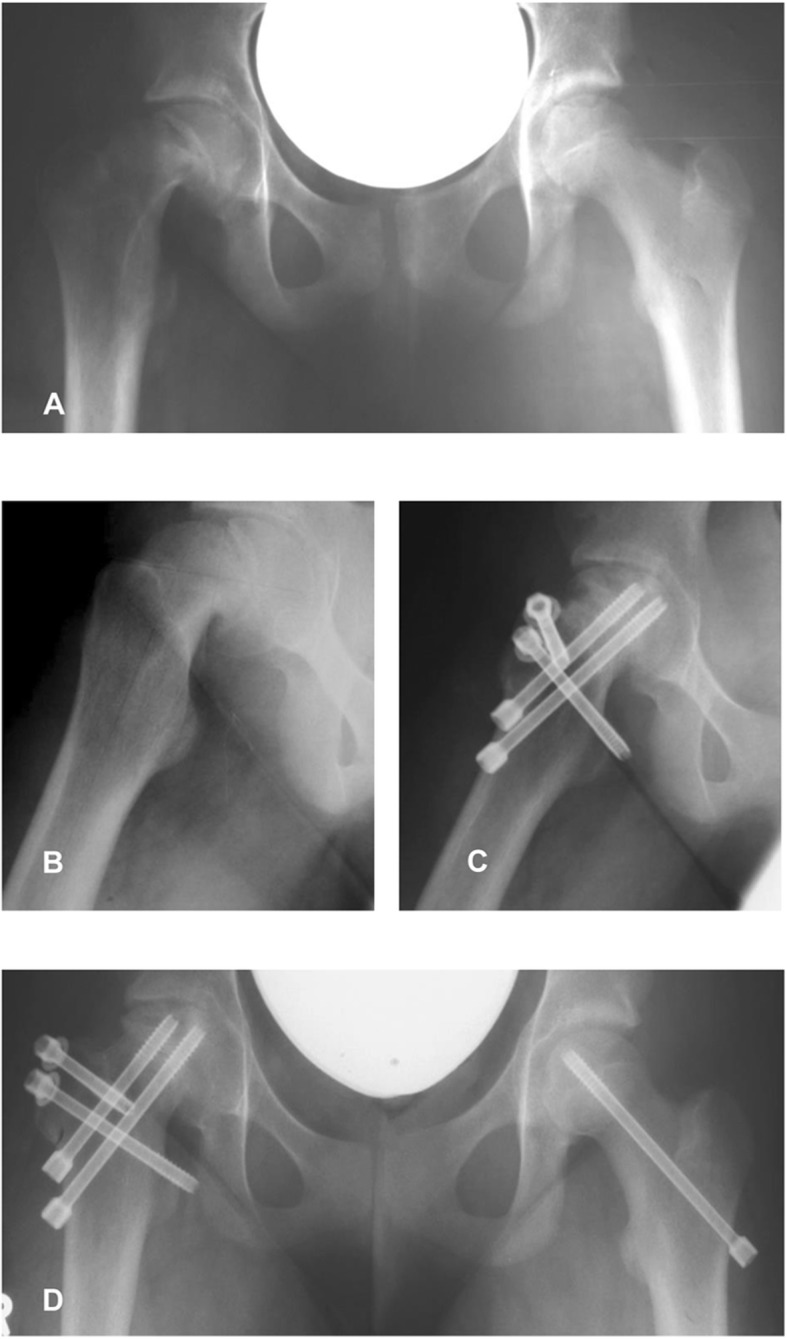
Preoperative (**a**, **b**) and 1 year postoperative (**c**, **d**) X-rays of a 12-year-old female patient with an acute on chronic SCFE with an SSA of 78°. This patient developed a partial avascular necrosis of the femoral head

At last follow-up, 4 of 15 patients (26%) had developed AVN of the femoral head (Fig. 2). Three of them were acute on chronic slips, and they all occurred during the early phase of our study (Table 2).

In total, we found complications in 6 out of 15 patients (36%). These patients had a DDC Score of II, III, or IV. As revision procedures, two early screw removals in patients with AVN followed by drilling of the necrotic bone were performed. Two patients had a leg length discrepancy which made an orthotic treatment necessary, and one of those patients had an AVN. Two patients, both treated in the early phase of our study, had joint instability (hip subluxation) after the modified Dunn procedure. One of them was treated by closed reduction and hip immobilization in a pelvic cast for 3 weeks. This patient developed AVN and had arthrodiastasis using an external fixation system 10 months after the modified Dunn procedure. The other patient had an open reduction with the removal of a loose body in the hip joint for the treatment of hip instability. An abduction splint was applied after surgery in this case.

## Discussion

Our results demonstrate that anatomic reconstruction is possible also in the most severe forms of SCFE if treated by the modified Dunn procedure. In 70% of the patients, the radiographic outcome is satisfactory or good, and the clinical outcome is even better, with 80% satisfactory results. Patients are less affected in quality of life even when radiographic and functional results were less satisfactory. There were also significant complications like AVN in 4 patients. We cannot finally say whether AVN occurred because of surgery or because of the slip itself because we did not perform perfusion MRI before and after surgery. The blood flow was tested before hip dislocation and after reduction by drilling of the femoral head with a K-wire, but this is not a safe method to ensure head perfusion. Upasani reported that the intraoperative assessment of head perfusion did not correlate with postoperative outcome [34]. Slongo, Ziebarth, and Huber described that the clinical stability of SCFE does not correlate with intraoperative stability [20, 25, 35]. The rate of AVN in our study is comparable to previous case series. However, in most studies, slips of various magnitudes were analyzed making comparisons with this study, including severe slips only, difficult. Most studies had lower AVN rates. Ziebarth and colleagues had no AVN in their study group with a follow-up of 1–3 years [19]. But Upasani et al. reported a complication rate of 37% and an AVN rate of 23% in their mixed patient cohort with a mean follow-up of 2.6 years [34]. In unstable slips, Sankar et al. noted an AVN rate of 26% after 22 months of follow-up [24]. The most common complication was revision surgery due to implant-related complications [19, 20, 33]. In our case series, those complications were not encountered. By now, arthrodiastasis using an external fixation system for 4 months combined with the drilling of the AVN region is usually used to treat AVN at our institution. These patients were mobilized with non-weight bearing of the affected hip for 6 months.

The modified Dunn procedure has also been advocated for unstable slips. Huber et al. outline the advantage of direct evaluation of epiphyseal perfusion during the procedure [35]. Tibor and Sink also suggest the modified Dunn procedure to be a good treatment option for acute and unstable slips [36]. Nevertheless, they reported a higher potential for complications than pinning in situ and a steep learning curve for the surgeon [36]. Sucato et al. recently stated in their review of patients treated by surgical hip dislocation that the average AVN rate for in situ pinning is 23.9% and for the modified Dunn procedure 16.7% when all studies looking at unstable SCFE are pooled [37]. Parsch et al. reported an AVN rate of 4.7% in their series of 64 patients with unstable slips, which were treated by capsulotomy, gentle reduction, and K-wire pinning [11].

The modified Dunn procedure can restore proximal femoral anatomy and hip function as shown in our study. Additionally, the intraarticular pathology can be evaluated and addressed at the time of surgery. Concerning the high rate of severe complications, we also use other surgical procedures for moderate stable slips, e.g., the Imhäuser osteotomy. Bali recently showed good results by combining the Imhäuser osteotomy with a femoral neck osteoplasty [38]. For the SCFE with a SSA greater than 50°, the intertrochanteric osteotomies should not be used, because of inacceptable postoperative deformity [1, 39].

The Dunn procedure is a good treatment option for those patients with severe chronic or acute on chronic slips. We saw that most of our AVN complications occurred in the early phase of our study, and we anticipate that complications like AVN will probably decrease with more experience. As we did not use MRI before surgery, we cannot definitely say whether the blood flow of the femoral head was compromised due to the slip or due to surgery in patients who developed AVN.

The limitations of this study are certainly the small number of patients, the retrospective character, and a missing control group. Additionally, the time of follow-up is different for each patient.

## Conclusions

The clinical and radiological outcome was good or satisfactory in most of the patients after modified Dunn procedure in this series. However, due to several limitations, conclusions have to be drawn with caution. Based on the results of this study, we believe that the modified Dunn procedure is only indicated if there is no other possibility to restore proximal femur geometry, as is the case in severe chronic or acute on chronic slipped capital femoral epiphysis. These slips cannot be repositioned and would lead to severe impingement, leg shortening, limited range of motion, and severe osteoarthritis [2, 9, 15, 40].

## Data Availability

The data (references) used to support the findings of this study are included within the article.

## Abbreviations

AVN: Avascular necrosis
NHP: Nottingham Health Profile
SCFE: Slipped capital femoral epiphysis
SSA: Southwick slip angle
